# Tuberculose mammaire: à propos d’un cas

**DOI:** 10.11604/pamj.2017.28.183.10742

**Published:** 2017-10-27

**Authors:** Tariq Bouhout, Badr Serji, Ebo Usman Egyir, Benyounes El amri, Imad Bouhout, Mehdi Soufi, Mohammed Bouziane, Tijani El harroudi

**Affiliations:** 1 Service de Chirurgie B, CHU Mohammed VI, Oujda, Maroc

**Keywords:** Tuberculose, sein, diagnostic, Tuberculosis, breast, diagnosis

## Abstract

Latuberculose mammaire est une affection rare. Elle pose un problème de diagnostic différentiel avec le cancer du sein du fait que la clinique et l'imagerie ne sont pas spécifiques. La tuberculose mammaire doit être évoquée surtout dans les pays endémiques ou chez le sujet immunodéprimé. Nous rapportons un cas de tuberculose mammaire chez une femme ménopausée pour soulever le problème de diagnostic qu'elle suscite.

## Abstract (en)

Breast tuberculosis is rare. It poses a problem of differential diagnosis because it may be mistaken for breast cancer as clinical examination and diagnostic imaging are not specific. Breast tuberculosis should be suspected especially in endemic countries or in immunosuppressed individuals. We report the case of a menopausal woman with breast tuberculosis in order to raise concern for diagnosis.

## Introduction

La tuberculose mammaire est une forme rare de la tuberculose extra-pulmonaire même dans les pays endémiques. Elle représente 0,06 à 0,1% de l'ensembledes localisations tuberculeuses [1,2]. La clinique et l'imagerie ne sont pas spécifiques de cette affection qui doit être distinguée des autres pathologies mammaires surtout les cancers mammaires pour éviter des investigations et des thérapeutiques parfois mutilantes. Nous rapportons un cas de tuberculose mammaire pour soulever le problème de diagnostic différentiel de la tuberculose mammaire avec le cancer du sein.

## Patient et observation

**Figure 1:**
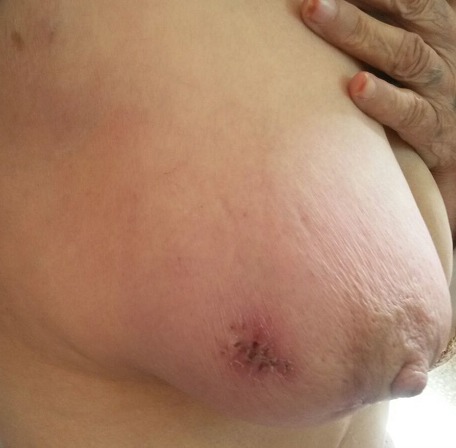
Vue de profil du sein droit

**Figure 2:**
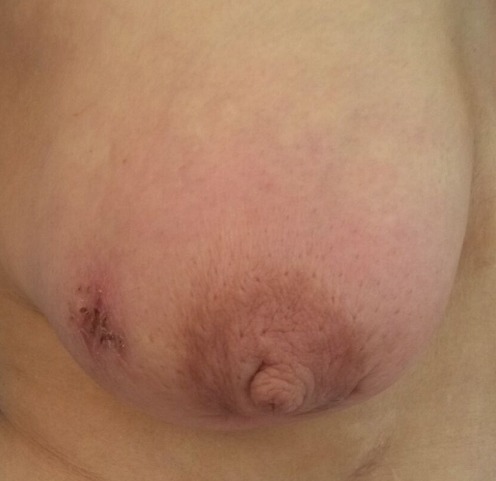
Vue de face du sein droit

**Figure 3:**
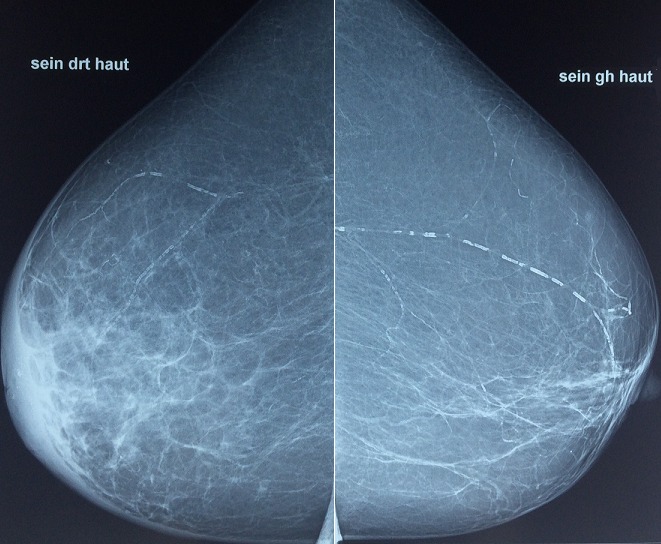
Mammographie du sein vue de profil

**Figure 4:**
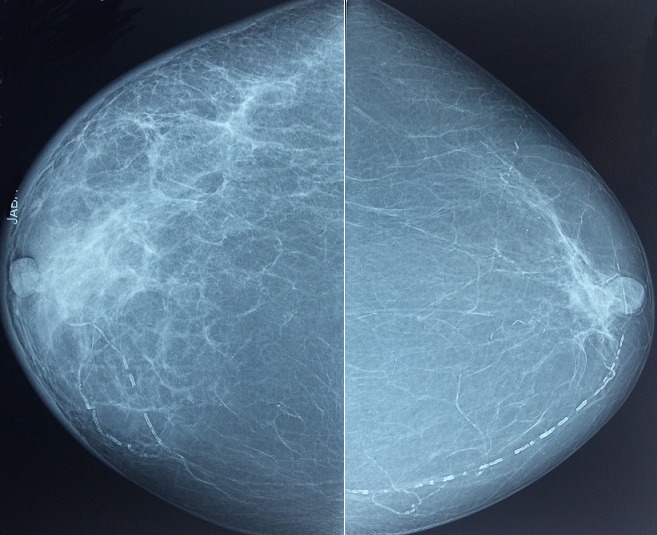
Mammographie du sein vue de face

Madame J.E, âgée de 60 ans, grande multipare, sans antécédents particuliers, notamment pas de notion de contage tuberculeux, qui s'est présentée à notre formation pour mastodynie apparu depuis deux mois. L'examen clinique a retrouvé un sein droit inflammatoire (Figure 1, Figure 2) avec présence d'adénopathies axillaires homolatérales évoquant un cancer du seinlocalement avancé, le sein gauche était sans anomalie de même le reste de l'examen clinique. La mammographie (Figure 3, Figure 4) a objectivé une accentuation de la densité mammaire droite avec épaississement diffus des parties molles cutanées sans lésions nodulaires ni microcalcifications. L'échographie mammaire a objectivé un épaississement cutané diffus avec un aspect infiltré des tissus mammaires surtout en retro-aréolaire, associé à de multiples adénopathies axillaires hypoéchogénes dontla plus grande mesure 40mm x 14mm .La lésion a été classé ACR 4. Une biopsie cutanée et une biopsie au Tru-Cut de l'adénopathie axillaire ont été réalisées, l'examen anatomopathologique a objectivé des remaniements fibro-inflammatoires chroniques non spécifique de la peau, et la présence de granulome épithélio-giganto-cellulaire avec nécrose caséeuse pour la biopsie de l'adénopathie axillaire. Le diagnostic d'une mastite tuberculeuse avec envahissement ganglionnaire associé a été retenu. La patiente a été adressée au centre de diagnostic et de traitement de la tuberculose pour un traitement anti tuberculeux.

## Discussion

La tuberculose mammaire est une forme très rare de tuberculose. Sa fréquence varie de 0,06% à 0,1% des atteintes tuberculeuses [1 ,2]. La rareté de cette forme clinique pourrait être expliquée par le fait que le tissu mammaire semble peu propice à la survie et à la multiplication du bacille tuberculeux [3]. Elle touche essentiellement la femme jeune [4]. La grossesse, la lactation et la multiparité sont des facteurs de risque [5], cela s'explique du fait de l'ectasie galactophorique au cours de la lactation. Les voies de contamination sont diverses [2]: la voie lymphatique ou une adénopathie axillaire est souvent trouvée; La voie hématogène, dans le cadre d'une milliaire tuberculeuse; Propagation par contiguïté à partir d'un foyer de voisinage; La voie canalaire: la dilatation des canaux galactophoriques chez la femme en période de grossesse ou d'allaitement augmente la sensibilité de ces canaux à l'infection par les bacilles; La voie directe: exceptionnelle, c'est la pénétration du bacille de Koch dans le sein à la suite d'une abrasion cutanée ou galactophorique. Classiquement, on distingue deux types de tuberculose mammaire : secondaire avec atteinte d'autres organes et primitive ou la tuberculose paraît strictement localisée au sein, cette dernière est la plus fréquente [3,6]. Concernant notre patiente l'atteinte était primitive. Sur le plan clinique, la tuberculose mammaire est caractérisée par l'absence de signes cliniques spécifiques [5], elle se présente soit sous la forme d'une masse nodulaire soit une masse inflammatoire mimant à un cancer du sein. Cependant, certains critères cliniques permettent d'orienter vers la tuberculose [2]: l'existence d'abcès récidivant rebelle aux antibiotiques ; l'existence d'adénopathies axillaires fistulisées et la fistule mammaire avec écoulement mamelonnaire. Sur le plan radiologique, il n'existe pas de signes mammographiques spécifiques de la tuberculose mammaire [7,8], la mammographie peut montrer des opacités hétérogènes irrégulières, mal limitée avec parfois des calcifications orientant plutôt vers une étiologie maligne. A l'échographie, la tuberculose mammaire apparaît souvent sous forme d'une image hypoéchogène, hétérogène bien ou mal limitée avec renforcement postérieur minime [8]. Le diagnostic de certitude est l'examen histologique [9] avec la mise en évidence de granulome épithéloide et giganto-cellulaire avec nécrose caséeuse. Le principal diagnostic différentiel à redouter devant la tuberculose mammaire est le cancer du sein,d'autres pathologies sont à discuter, comme l'abcès du sein, le fibroadénome, la sarcoïdose et les mastites granulomateuses. Dans notre observation, devant un sein inflammatoire chez une femme âgée et ménopausée, le premier diagnostic à évoquer est essentiellement le cancer du sein et seul l'examen histologique qui a permis de poser le diagnostic de tuberculose mammaire. La prise en charge thérapeutique de la tuberculose mammaire repose sur les antibacillaires, avec un éventuel drainage percutané d'un abcès du sein ce qui n'était pas le cas chez notre patiente, la chirurgie (mastectomie) peut être proposée en cas de résistance au traitement médicale [5].

## Conclusion

La tuberculose mammaire est une localisation très rare, même dans les pays d'endémies. Les tableaux cliniques et radiologiques sont trompeurs, et posent un problème diagnostic en particulier avec le cancer du sein. L'examen anatomopathologique reste le principal élément pour le diagnostic de certitude.

## Conflits d’intérêts

Les auteurs ne déclarent aucun conflit d'intérêts.
